# Quality of Life and Mental Health Problems in Pediatric Cardiac Arrest Survivors

**DOI:** 10.3390/children12101397

**Published:** 2025-10-16

**Authors:** Tina Schwartz, Michael Weidenbach, Ingo Dähnert, Christian Paech, Franziska Markel

**Affiliations:** 1 Faculty of Medicine, University Leipzig, Augustusplatz 10, 04109 Leipzig, Germany; 2 Heart Center Leipzig, University Hospital Leipzig, Strümpellstraße 39, 04289 Leipzig, Germany; michael.weidenbach@helios-gesundheit.de (M.W.); ingo.daehnert@helios-gesundheit.de (I.D.); christian.paech@helios-gesundheit.de (C.P.); franziska.markel@helios-gesundheit.de (F.M.); 3 Department of Congenital Heart Disease—Pediatric Cardiology, Deutsches Herzzentrum der Charité, Augustenburger Platz1, 13353 Berlin, Germany; 4 Deutsches Herzzentrum der Charité, Department of Psychocardiology, Department of Developmental Pediatrics, Augustenburger Platz 1, 13353 Berlin, Germany

**Keywords:** pediatric resuscitation, cardiac arrest, IHCA, congenital heart defects, quality of life, mental health, ECMO, behavioral problems, aftercare programs, follow-up, standardized care

## Abstract

**Highlights:**

**What are the main findings?**

- Out of 127 pediatric cardiac arrest (CA) patients with heart disease, 72% survived to hospital discharge, and 22 are receiving follow-up care in an outpatient clinic.
- Overall quality of life was comparable to healthy peers, but children with longer resuscitation time or multiple resuscitations showed lower scores in physical, emotional, social, and school functioning.
- A total of 21% of children showed elevated emotional and hyperactivity-related difficulties (based on parent reports), and peer problems (based on self-reports), indicating increased psychological stress.
- One-third of school-aged survivors attend special needs schools, highlighting potential neurodevelopmental and academic impact.

**What is the implication of the main finding?**

- While survival and average quality of life appear reassuring, a substantial proportion of survivors face subtle but relevant emotional, behavioral, and academic challenges.
- These findings underscore the urgent need for long-term follow-up programs with routine neurodevelopmental and neuropsychological screening to support at-risk pediatric cardiac arrest survivors.

**Abstract:**

**Background:** Current research is paying more attention to neurological outcomes and quality of life after life-threatening events. Children with heart disease are particularly vulnerable, especially after resuscitation events. While newer data show that adults with heart failure and a left-ventricular assist device suffer from a higher incidence of depression, mental health in pediatric heart disease patients is poorly understood. This is the first study in Germany to examine the quality of life and psychological burden in cardiac arrest survivors with congenital or acquired heart disease. **Methods:** This monocentric study retrospectively analyzed survival outcomes of pediatric heart disease patients who underwent in-hospital resuscitation between 2008 and 2022. The PedsQL and Strength and difficulties questionnaires were prospectively administered to survivors to assess quality of life and emotional/behavioral problems, while academic achievements were additionally documented. **Results:** Of 127 patients experiencing cardiac arrest, 91 (71.7%) survived to discharge. Most had complex congenital heart diseases; mean cardiopulmonary resuscitation duration was 14 min. Five patients received extracorporeal cardiopulmonary resuscitation. Of the 22 patients who were receiving follow-up care at the pediatric cardiology outpatient clinic at the time of the study, 14 completed questionnaires were received. Overall quality of life was comparable to healthy controls, though those with prolonged or multiple resuscitations showed lower physical, emotional, social, and school functioning scores. The Strengths and Difficulties Questionnaire revealed no pathological scores but elevated average values for hyperactivity and emotional problems in parent reports, and emotional and peer difficulties in self-reports, indicating increased psychological burden. **Conclusions:** While survival rates are comparable to international data, gaps exist in structured follow-up and neuropsychological care, especially for high-risk subgroups like ECMO survivors. Routine neuropsychological screening and multidisciplinary outpatient programs are essential to improve long-term follow-up care.

## 1. Introduction

To date, there is a paucity of data on in- and out-of-hospital resuscitations of infants, children, and adolescents in Germany. Based on data from the USA and Japan, we can assume roughly 5000 resuscitations per year for Germany, Austria, and Switzerland [1]. Despite emerging suggestions from international guidelines, there is no standardized follow-up care for pediatric cardiac arrest survivors yet.

International data on the incidence of cardiovascular arrest (CA) in children vary widely in the literature. However, all show that resuscitation events occur up to three times more frequently in patients with heart defects, especially in the immediate postoperative course after cardiac surgery compared to other pediatric subdisciplines [2].

Despite the presence of varying incidences of in-hospital cardiac arrest (IHCA), there is also evidence of significant disparities in survival rates and risk factors. A recent survey of the Pediatric Life Support working group of the European Resuscitation Council demonstrated a survival to hospital discharge rate of 32–57% after pediatric IHCA in European countries [3].

In their study of 250 pediatric patients who suffered cardiac arrest in the pediatric intensive care unit, Del Castillo et al. found a survival rate of 40.4% after resuscitation measures until discharge. In addition, the study revealed that patients with pre-existing cardiac conditions had higher survival rates compared to those with other non-cardiac diseases [4]. In their prospective multicenter study in Spain, Portugal, Italy, and Latin American countries, the neurological status of 88 patients (87%) was assessed at hospital discharge. Of these patients, 73.8% demonstrated either normal function, mild impairment, or no change from their condition compared to pre-event score. Subsequent to a one-year period, 65 patients underwent neurological follow-ups, which revealed that 81.5% exhibited no or only mild disabilities.

Meert et al. studied data from 329 children suffering from IHCA, analyzing risk factors influencing patient outcome. They demonstrated that patients with extracorporal cardiopulmonary resuscitation (ECPR) therapy had lower Vineland scores, indicating poorer neurofunctional outcome [5].

In their study on quality of life (QoL) of patients following total cavopulmonary connection (TCPC) palliation, Brosig et al. found consistently lower mean scores across nearly all Pediatric Quality of Life Inventory (PedsQL) domains in the TCPC cohort compared to healthy peers. Within their cohort children after three-staged palliation had lower scores compared to children with a two-staged palliation highlighting the influence of surgical procedures and hospitals stays on the QoL [6].

A review by Meentken et al. [7] indicates that a range of 12–31% of children with CHD undergoing cardiac surgery develop post-traumatic stress disorder (PTSD). However, these findings are comparable to the results of hospitalized children without CHD. Hospitalization, emergency department visits, intensive care admissions, and medical interventions all increase the risk of psychological distress in addition to the physical health concerns.

Furthermore, families with children with CHD experience a high level of family life stress, as has been demonstrated in a study by Cassedy et al. [8]. Within this cohort, greater severity of CHD was associated with elevated levels of chronic disease-related stress in patients and parents long after surgery. Both disease-related and family life stress have also shown an effect on the behavioral and emotional function of the patients in this study cohort. It seems that children with severe heart disease are particularly vulnerable, and the psychological impact of survival following resuscitation, along with the subsequent consequences, serves to exacerbate an already elevated level of stress.

Thus, in addition to long-term data on the neurological outcome, patient-related outcome measures are becoming increasingly important. Standardized questionnaires are utilized to evaluate patient-related health, wherein patients self-report their health status, quality of life, or treatment outcomes [9]. The Pediatric Core Outcome Set after Cardiac Arrest constitutes an advisory statement from the International Liaison Committee on Resuscitation [10], which suggests a data collection regarding the patients’ QoL subsequent to the event. Notwithstanding the aforementioned recommendations, mere three registries that provided responses to the European survey collected data on the QoL of patients beyond 30 days following the event [10].

A preliminary systematic study on the long-term emotional and behavioral outcome of survivors of CA in childhood was conducted by Van Zellem et al. [11]. Despite the absence of statistically significant differences between the patient group and the control group with regard to levels of anxiety, depression, or post-traumatic problems, a higher prevalence of attention problems has been diagnosed in the patient group. They demonstrated that deficits in emotion and behavior significantly impact children, their families, and society. They emphasized the need for neuropsychological aftercare.

To the authors’ knowledge, no survey has hitherto been conducted on the emotional and behavioral outcomes of children suffering from congenital, acquired or arrhythmogenic heart disease who have survived CA in Germany. For multifactorial reasons in, the international data cannot be transferred to German hospitals with sufficient validity. We hypothesized that children with cardiopulmonary resuscitation, especially those with long resuscitation times, multiple resuscitations, and severe heart defects are likely to exhibit mental health problems and face an elevated risk of developing such conditions when compared to children without such medical histories.

The present study investigates the epidemiology of in-hospital cardiac arrest in children with heart disease in a German heart center. Secondly, the objective of this study is to concentrate on patient-reported outcome measures of individuals who have survived CA. All statistical analyses were conducted using descriptive methods only.

## 2. Materials and Methods

### 2.1. Study Design and Patient Population

This study was conducted at the Department of Pediatric Cardiology of the Heart Center Leipzig, Germany. It was reviewed and approved by the Ethics Committee Leipzig on 24 July 2024 (211/24-ek). All patients or their parents gave their written consent. This study was conducted in compliance with the Declaration of Helsinki.

In this monocentric study, we retrospectively enrolled all patients aged 0–18 years with congenital or acquired heart disease who were resuscitated at the Heart Center Leipzig due to IHCA in the period from January 2008 to December 2020. For the prospective survey, survival to date was a further inclusion criterion.

Inclusion criteria:

- congenital or acquired heart disease
- age 0–18 years
- IHCA
- return of spontaneous circulation (ROSC)
- survival to date
- regular follow-up visits in the outpatient clinic

We conducted a search via the electronic hospital information system, initially including all patients with the ICD-Codes for “respiratory arrest”, “cardiopulmonary arrest”, “cardiac arrest”, “successful resuscitation”, “without successful resuscitation”, “ventricular flutter and fibrillation”, “pulseless electrical activity”, “peripheral circulatory failure” and the OPS-Codes for “measures in the context of resuscitation”, “cardiac or cardiopulmonary resuscitation”, “operative resuscitation”. Keywords from the diagnosis list and the epicrisis were used for the second data query: “post-resuscitation”, “post cardiopulmonary resuscitation”, “post-circulatory arrest”, “post-cardiac arrest”, “post-cardiac massage”, “after resuscitation measures”, “exitus letalis”. The detailed search terms and codes can be found in Appendix A.

### 2.2. Data Collection

The data were recorded from routine clinical documentation systems of the Heart Center Leipzig, in accordance with the Utstein Guidelines [12,13,14]. This comprised demographical data (including age, weight, length, gender) as well as clinical data, such as diagnoses, duration of hospitalization, or intensive care therapy, in addition to resuscitation data, such as the duration of the resuscitation, the necessity for extracorporeal membrane oxygenation (ECMO)-therapy, and arterial and venous blood gas analyses including levels of lactate, pH value and base excess, and at least the type of education. The Pediatric Cerebral Performance Category Scale (PCPC) was retrospectively assigned based on the patients’ medical history and clinical course. According to Warnes et al., we divided the patients in three subgroups as follows: simple (e.g., Aortic valve stenosis), moderate (e.g., Ventricular septal defect, Coarctation of the aorta) and complex (e.g., Single ventricle, Tetralogy of Fallot) congenital heart disease (CHD) [15].

### 2.3. Assessments

All individuals who met the inclusion criteria have been contacted via telephone between October 2024 and February 2025 in a prospective manner. Following the formal consent process, the participants were sent study questionnaires via post within a four-week period. Following a period of 8 and 12 weeks, the parents were contacted via telephone in order to provide a reminder. The test results were compared with the results of a large healthy, pediatric cohort of the LifeChild-project in Eastern Germany [16] as well as with the results of patients with univentricular heart and Fontan palliation [17]. The participants did not receive financial compensation.

#### 2.3.1. The Pediatric Cerebral Performance Category Scale (PCPC)

The PCPC is a widely utilized instrument for the assessment of functional morbidity and cognitive impairment subsequent to brain injury or critical illness. The patient’s condition may be evaluated using a six-point scale, categorizing states as follows: normal, mild disability, moderate disability, severe disability, coma, and death [18,19].

#### 2.3.2. Pediatric Quality of Life Inventory (PedsQL)

The Pediatric Quality of Life Inventory was utilized to verify the quality of life post-resuscitation. The generic core scale includes 23 items, encompassing physical health, emotional functioning, social, and school functioning. The questionnaire is administered in two forms as follows: as a parent proxy report and as a self-report from the age of five years. In the telephone conversation, it was clarified whether the child was able to independently complete the questionnaire themselves or if assistance would be required from the parents. The evaluation of the questionnaire employed a 5-point Likert scale. The scale ranges from 0 (never) to 4 (almost always). The items are evaluated using a reverse 0–100 scale: for example, 0 corresponds to 100, 1 to 75, 2 to 50, 3 to 25, and 4 to 0. It is evident that achieving a high score is indicative of an enhanced QoL. The total score can be calculated and the mean for each subgroup can be determined. An analysis of scores can be conducted at the level of individual items, the scale as a whole, and the summary level [20].

#### 2.3.3. Strengths and Difficulties Questionnaire

The Strengths and Difficulties Questionnaire (SDQ) was developed to record positive and negative behavioral characteristics of children. The instrument poses questions regarding a range of relevant aspects of life, including prosocial behavior, hyperactivity, emotional symptoms, conduct problems, and peer relationship problems. The final score is then categorized as normal, borderline, or abnormal. The administration of the questionnaire occurs in two forms: as a parent proxy report and as a self-report from the age of four years. During the telephonic interactions, the researchers ascertained whether the child was capable of completing the questionnaire independently or if assistance would be needed from the parents. The evaluation of the subscales is achieved through the allocation of scores to each subgroup ranging from 0 to 2, depending on whether it is “not true”, “somewhat true”, or “certainly true”. The Total Difficulties Score (TDS) is the average of four subgroups, with the exception of prosocial behavior. The prosocial behavior score is reported separately as a measure of strengths. Higher TDS has been demonstrated to be indicative of greater difficulty; conversely, higher prosocial scores have been shown to correlate with more positive social behaviors. The score is divided into three categories “normal”, “borderline”, and “abnormal” [16,21,22].

### 2.4. Statistical Analysis

All statistical analyses were conducted using descriptive methods [23]. Due to the limited sample size of the patient cohort, it was not feasible to calculate meaningful *p*-values or draw conclusions regarding statistical significance. Instead, we focused on providing a transparent and comprehensive overview of the observed data. This approach was chosen because, with such a small cohort, the calculation of *p*-values and the interpretation of statistical significance could lead to misleading or unreliable conclusions. To avoid any potential misinterpretations, we refrained from conducting inferential statistical tests and limited our analysis to basic summary measures such as means and standard deviations.

## 3. Results

**Figure 1:**
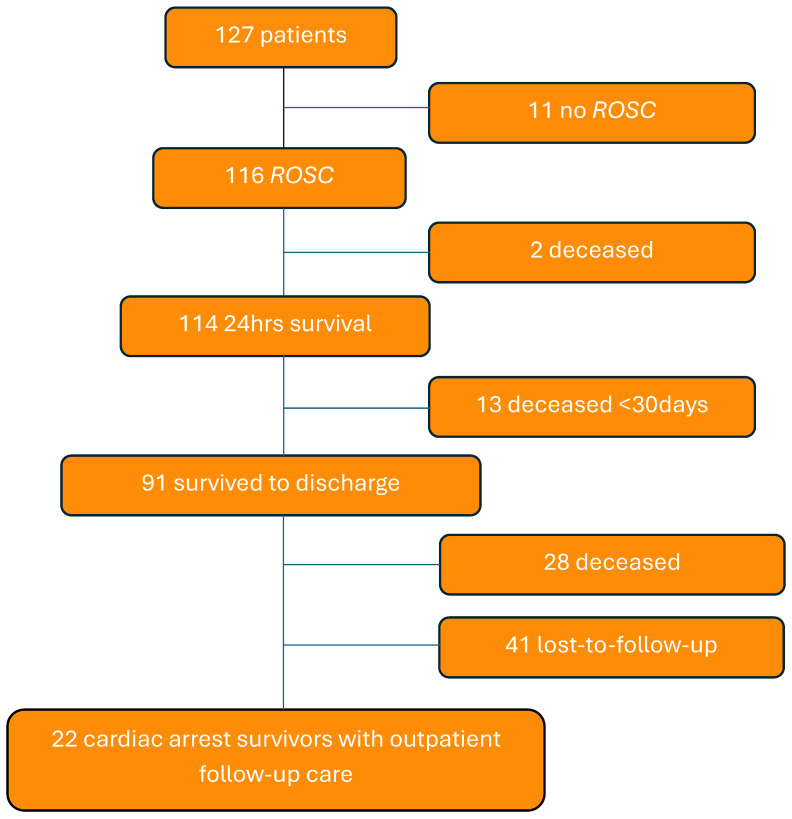
Flowchart of study population (ROSC = return of spontaneous circulation).

**Figure 2:**
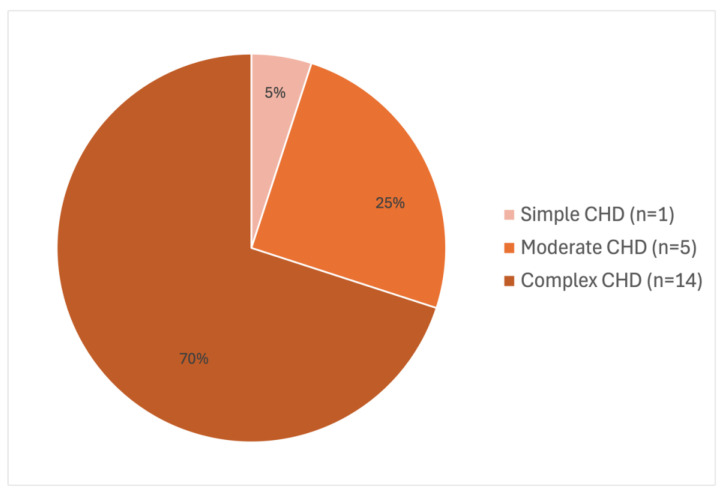
Distribution of congenital heart defects (CHD) complexity levels in the cohort [15]. In our cohort 14 patients had complex CHD (2 TGA,1 DORV, 1 pulmonary atresia, 5 univentricular heart, 2 Truncus arteriosus communis, 2 TOF, 1 Shone Komplex); 5 patients had Moderate CHD (3 AVSD, 1 VSD, 1 Aortic coarctation); 1 patient had Simple CHD (Aortic stenosis). Two patients had acquired heart disease (Myocarditis, Long-QT) (TGA = transposition of the greater arteries, AVSD = Atrioventricular Septal Defect, DORV = Double Outlet Right Ventricle, VSD = Ventricular Septal Defect, TOF = Tetralogy of Fallot).

A total of 127 patients suffered from CA and underwent cardiopulmonary resuscitation at Heart Center Leipzig. The mean age of the patients examined was 1.74 (±3.726) years old. A total of 43.5% were female, 56.5% male. Within our cohort, 91 patients survived to discharge (71.7%). Figure 1 illustrates the criteria used to select our final cohort of 22 patients. As demonstrated in Figure 2, the distribution of CHD reveals that the majority of patients had a complex congenital heart disease. Two out of twenty-two patients had an acquired heart defect (Long-QT-Syndrome, Myocarditis). One of them answered the questionnaires. The mean duration of resuscitation was 14.06 (±20.1) minutes. A blood gas analysis was available from 15 patients during the resuscitation process and showed an average pH of 7.23 (±0.2) and an average maximal lactate of 7.3 mmol/L (±6.9). Five patients were supported with an ECPR. The results of the resuscitation evaluation are shown in Table 1.

In most cases, it was not possible to collect the PCPC score retrospectively in a way that could be considered adequate, due to missing data. For this reason, we refrained from listing single values for individual patients.

**Figure 3:**
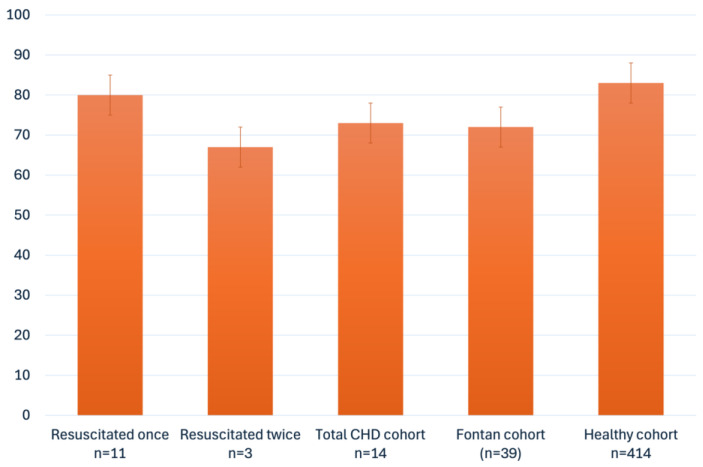
PedsQL mean score in comparison with healthy [20] and Fontan cohort [6].

**Figure 4:**
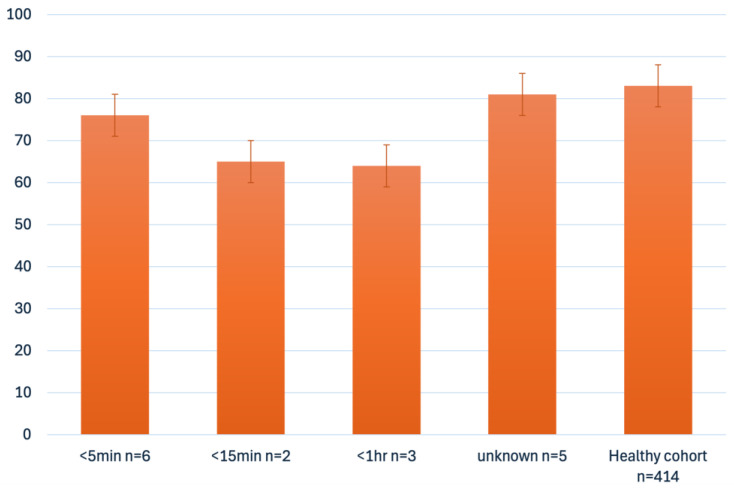
PedsQL mean score depending on resuscitation duration and in comparison with healthy cohort [20].

This study also prospectively examined the quality of life and mental health problems in the cohort of CA survivors. From 22 questionnaires distributed, we received 14 back for evaluation. Analysis of the PedsQL questionnaires revealed that children with heart disease with a history of cardiopulmonary resuscitation have a quality of life similar to that of healthy controls [20] (Figure 3). Within our cohort, patients who had experienced multiple resuscitation events (n = 3, 21%, Figure 3) and those with a longer resuscitation time (n = 5, 36%, Figure 4) tended to have a poorer quality of life. In comparison with a healthy cohort these patients demonstrated reduced QoL in the subgroups physical health, emotional functioning, social functioning, and school functioning (Table 2) [20].

Evaluating the SDQ, we found that our patients showed no difficulties in any of the subdomains (Table 3). While the participants did not exceed the established limit values, the average values were higher than those in a healthy cohort. In the parent reports, the values for hyperactivity (5.8 ± 0.7) and emotional problems (2.6 ± 1.8) are particularly elevated. Difficulties in the hyperactivity domain were observed in 3 out of 14 children (21%), while emotional problems were identified in another 3 children (21%). Based on the self-reports (8 of 14 children), slightly elevated mean scores were observed for emotional problems (3.75 ± 2.1) and peer relationship problems (3.1 ± 0.8). Notably, two children demonstrated pronounced difficulties in peer relations.

In addition to the mean scores, individual responses revealed that 3 out of 14 children (21%) experienced challenges related to hyperactivity, while another 3 (21%) had difficulties coping with emotional problems. Of the children who completed the self-questionnaire (8 out of 14), 2 children had noticeable values for problems with peers.

Evaluating the results regarding the schooling of school-age children, we found that 6 out of 18 children attend a special needs school.

## 4. Discussion

This is the first study in Germany to examine the quality of life and psychological burden in cardiac arrest survivors with congenital or acquired heart disease. Of 127 patients, 72% survived to hospital discharge, most of whom had complex congenital heart defects. Overall quality of life was comparable to that of healthy controls; however, patients who underwent prolonged or repeated resuscitation reported lower physical, emotional, social, and school functioning. A psychological screening tool showed no pathological findings, but elevated levels of hyperactivity and emotional difficulties indicated an increased psychological burden. The study also revealed significant gaps in structured follow-up care. Therefore, routine neuropsychological screening and multidisciplinary follow-up programs are strongly recommended to close this knowledge gap, enable the prompt initiation of therapeutic interventions, and have the potential to positively impact long-term outcomes.

### 4.1. Outcome

In our cohort 91.3% of the patients with cardiac arrest and subsequent cardiopulmonary resuscitation had ROSC and 72% of patients survived until hospital discharge. The survival rates after in-hospital cardiac arrest vary greatly in the literature.

The Pediatric Life Support working group of the European Resuscitation Council recently investigated the epidemiology of pediatric cardiac arrest in Europe. They collected data from 33 European countries. Only 13 of these countries have an active pediatric registry. Within these registries ROSC rates after pIHCA ranged from 60% to 71% and survival to hospital discharge or 30-day survival ranged from 32% to 58% [2]. The results of our small cohort are comparable with the previous data. In a large multicenter study in Europe and Latin America by del Castillo et al., the survival rate of pediatric patients with IHCA was 69.1%. Survival to discharge was described with 40.4% in this cohort. However, del Castillo et al. analyzed a cohort of 250 children during their stay on a general PICU. Within their cohort, they described that pediatric patients with cardiological diseases had a higher survival rate after CA than hemato-oncologic or general pediatric patients [3].

### 4.2. Resuscitation Data Quality

In the course of evaluating the resuscitation data we aimed to report the dataset recommended within the Utstein Guidelines. However, the collection of these parameters posed significant challenges, as the documentation at the time of resuscitation was often inadequate or missing, and the archiving of patient records was frequently incomplete. Therefore, no sufficient PCPC score for the CA survivors could be determined.

The first step in building a registry is for correct data to be collected and documented in a standardized manner. However, it is considered an important obstacle that must be overcome for success [24,25].

### 4.3. Follow-Up After Cardiac Arrest

International resuscitation guidelines recommend a thorough neurological assessment during the post-resuscitation care in order to provide informed decision making based on neuroprognostication using different assessments, such as the Glasgow Coma Scale, PCPC score, reflex testing, pupil reactivity to light, electroencephalography, laboratory parameters, or imaging procedures. If there are new neurological symptoms, urgent neuroradiologic imaging is required [26,27].

European countries are requested to participate in cardiac arrest registries to improve the understanding of epidemiology and resuscitation outcomes in Europe [27,28]. Furthermore, a standardized and structured follow-up after cardiopulmonary resuscitation is recommended [28]. After ECMO- support, the Euro-ELSO (Extracorporeal Life Support Organization) guidelines stipulate that a structured follow-up should begin even before discharge and include a detailed neurologic evaluation, magnetic resonance imaging (MRI) scan of the brain for those who suffered, e.g., a cardiac arrest, a plan for routine, pediatric care to establish follow-up, neurodevelopmental assessment, nutritional assessment and dietetic plan, psychologic, and social support [28,29].

Our high rate of patients lost-to-follow-up (31.5%) means a severe lack of follow-up structure. Only 4 of our 22 patients received a structured neuropsychological treatment in a specialized care center. For the five ECPR patients no information was found in the documentation system in four cases, and no structured neuropsychological care is established in one case [17]. As these patients represent the most vulnerable group, a multi-disciplinary follow-up care is critical [30].

In accordance with our findings, Cvetkovic et al. revealed that only 37.6% of pediatric patients receiving ECMO underwent a neurological assessment prior to hospital discharge in their study among 211 European ECMO-centers. A structured longitudinal follow-up, defined by regular, pre-scheduled assessments, was in place for only 15 individuals (11.2%), whereas the majority of 99 individuals (74.4%) did not have any formal follow-up [31].

### 4.4. Quality of Life

Given the numerous risk factors present in our patient cohort, it is challenging to conceive that their quality of life is only marginally different from that of healthy children. Nevertheless, other studies have also demonstrated that the quality of life of children after intensive care unit hospitalization is comparable to that of healthy controls [29].

In their review, Huebschmann et al. concluded that the quality of life varies greatly among individuals [32]. The reasons for this variation were numerous. The identified risk factors were age at the time of resuscitation, PCPC at discharge, use of ECMO, high lactate levels, and parental mental health issues.

Also, resilience is an important tool to deal with possibly life threatening situations and plays a critical role in helping children adapt to chronic illness [33]. It supports emotional well-being, treatment adherence, and quality of life. Strong family and peer support enhance resilience, making it a key factor in long-term adjustment and healthy development. Although resilience in CHD patients is significantly lower [34], our assumption, that quality of life decreases with increasing severity of heart defects, has not been confirmed in our small cohort.

We found that patients who had experienced multiple resuscitation events and those with a longer resuscitation time tended to have lower values for quality of life compared to others within our cohort.

Matos et al. also found that, in a general pediatric study, that the longer the CPR was performed, the lower the quality of life [35]. During the first 15 min of CPR, the survival rate decreases by 2.1% with each passing minute, and the likelihood of a favorable neurological outcome decreases by 1.2% for every minute. The authors hypothesize that surgical cardiac patients exhibit the highest adjusted odds ratios for survival and favorable neurological outcomes compared to children with non-cardiac diseases and a cardiopulmonary resuscitation in a non-surgical setting.

Still, patients with a history of cardiac surgery are at an increased risk of developing neurodevelopmental difficulties at some point in their lives. These difficulties may manifest as impairments in motor function, cognition, language, and/or other domains [33]. Brossard-Racine et al. found that patients with complex CHD had reduced total and regional brain volumes, as well as microstructural alterations, which are frequently linked to lower performance in specific cognitive domains, such as executive functioning and memory [18].

### 4.5. Psychological Burden

A parallel can be drawn when examining the SDQ. Although the results do not exceed any limits, they are still elevated compared to the results of healthy children. The observed trend should nevertheless alert us to the potential for future difficulties. As van Zellem et al. also confirm, the possibility of a long-term problem is substantiated. Despite still normal levels for anxiety, depression, or post-traumatic stress symptoms, a greater prevalence of attention problems was noted in their study [11].

A further study of 145 cardiac arrest survivors found no elevated levels of anxiety or depression. Within their cohort, the authors observed more difficulties in older patients demonstrating more externalizing and attention problems. The average age of our cohort is 1.74 years (±3.726), and the average age at the time of the survey was only 12.4 years (±6.7). This could explain why our small group still displays normal values. Problems may only become apparent later during adolescence or adulthood [11].

One possible explanation is that the questionnaires we received came primarily from children in stable family environments, which represents one less risk factor for mental health issues. Despite multiple contact attempts with all families, it is likely that we were unable to reach those children who may be experiencing difficulties or come from families under significant stress.

When evaluating the SDQ, the discrepancy between the healthy cohort [16] and the self-report is less pronounced than the discrepancy between the parent-report and the healthy cohort. This finding suggests the presence of divergent perceptions within the family unit and highlights the need for a comprehensive understanding of each patient’s unique social role. To ensure the comparability of the results obtained from the older children, it would be advisable to use both self- and parent-report methodologies. This should be investigated further in future studies. For younger children, though, parental external assessment is a key component in our methodology. Parents often rate their child with poorer scores, as has been widely documented [11]. Jaschinski et al. had referred to this as the so-called “vulnerable child syndrome” [17,36]. Moreover, this group has determined that the necessity for one or more cardiac surgeries constitutes a risk factor for negative psychosocial impacts on the patient’s social environment within five years of surgery [36]. When considering the specific risk factors (e.g., multiple hospital stays, traumatic procedures, fragile family structure, neurodevelopmental disorders, PTSD) associated with heart diseases in children, it is crucial to enhance our ability to identify these factors and to organize outpatient care to ensure patients receive optimal treatment and preventive support, thus preventing the development of more severe psychological complications [7].

### 4.6. Type of Education

Finally, we note that 6 out of 18 (33.3%) children attend a special needs school. The reasons for this are varied and would need to be investigated separately. Chronically ill children have more frequent hospital stays or doctor’s appointments, resulting in learning deficits and social isolation. Schmitt et al. compared the school education of CHD patients with and without trisomy 21 in a study of 2873 children. A total of 28.4% of the CHD patients without trisomy 21 reported having ever received special education services [37]. The most psychological disorders for the non-trisomy 21 group were learning and language disorders, developmental disability, attention, and anxiety disorders.

Geva et al. [38] demonstrated that deficits in language, memory, and academic skills are associated with structural brain changes, including reduced volumes in the hippocampus, thalamus, and caudate, as well as lower white matter integrity in key tracts.

### 4.7. Recommendations

This is the first study to report data on the quality of life and psychological burden experienced by pediatric cardiac arrest survivors in Germany. Our data indicate that we are in need of quality improvement initiatives and structured multi-disciplinary follow-up-clinics of cardiac arrest survivors. Standardized screening tests assessing mental health problems should be introduced at certain time points to ensure an adequate diagnosis and therapy, as early as possible. As the P-COSCA already suggests for studies regarding p-CA, we should also aim to use PedsQL-questionnaires gathering data on quality of life of this patient cohort in a standardized matter. Patients should be discharged with scheduled follow-up appointments, during which the questionnaires should be repeated to monitor any changes or emerging dynamics over time.

### 4.8. Limitations

This is a single-center study with a small cohort. Due to the retrospective study design, some desired data are unavailable because they were missing from the patient documentation. Additionally, the outcome in a relevant patient cohort remains unknown due to a high percentage of patients lost to follow-up. Furthermore, there is a suspicion of bias, as it is primarily parents who demonstrate optimal childcare practices that complete the questionnaires. We cannot distinguish whether patient-reported outcome measures are related to the resuscitation event or the severe course of the illness itself. Due to the limited sample size, we opted against conducting a statistical analysis. To enhance the significance of the findings, it is imperative that studies encompassing a more substantial sample size be undertaken.

## Figures and Tables

**Table 1:** Resuscitation values. (✓ = Data available).

	Heart Disease	Resuscitation Time (Minutes)	ECPR	Type of BGA	Minimum pH Value	Minimum Base Excess mmol/L	Maximum Lactate mmol/L	QoL Could Be Evaluated	Type of Education
1	Aortic stenosis	35	-	n/a	n/a	n/a	n/a	✓	Special education
2	TGA	15	-	2	7.33	−3.8	2.6	✓	Regular school
3	TGA	2	-	2	7.4	−0.6	3.2	✓	Regular school
4	AVSD	2	-	2	7.4	−7.5	2.4	-	Special education
5	DORV	n/a	-	n/a	n/a	n/a	n/a	✓	Regular school
6	AVSD	n/a	-	n/a	n/a	n/a	n/a	-	Special education
7	Perimyocarditis	n/a	✓	2	7.4	−1.5	3.5	✓	Regular school
8	Long QT Syndrom	5	-	2	7.2	−7.8	4.3	-	Regular school
9	VSD	2	-	2	7.1	−12	16	✓	Regular school
10	Pulmonary atresia	n/a	-	2	7.3	−4.7	5.3	✓	Regular school
11	Hypoplastic right heart	10	-	2	7.3	−3.7	3.1	-	Regular school
12	Univentricular heart	n/a	-	1	7.1	3.1	2	-	Special education
13	Truncus art. comm.	1	-	2	7.0	−18	21	✓	Regular school
14	TOF	75	✓	2	7.36	−3.7	3.1	-	Regular school
15	AVSD	2	-	2	7.29	−0.8	1.1	✓	Regular school delayed
16	Shone Komplex	10	-	n/a	n/a	n/a	n/a	-	Nursery school
17	TOF	1	-	n/a	n/a	n/a	n/a	✓	Nursery school
18	Univentricular heart	n/a	✓	n/a	n/a	n/a	n/a	-	Nursery school
19	Truncus art. comm.	n/a	✓	n/a	n/a	n/a	n/a	✓	Nursery school
20	Univentricular heart	7	-	n/a	n/a	n/a	n/a	✓	Special education
		40	-	n/a	n/a	n/a	n/a	-	
21	Hypoplastic left heart	n/a	-	2	7.3	−8.2	2.1	✓	Regular school
		n/a	✓	2	6.6	−31	18.0	-	
22	Aortic coarctation	2	-	2	7.3	−1.5	13.6	✓	Special education
		16	-	2	7.3	−3.7	16.0	-	

**Table 2:** PedsQL Mean Scores of children with heart disease and CPR in comparison with healthy cohort [20].

	Children with Heart Disease (n = 22)	Healthy Cohort (n = 401)
Total score	77.3 (±2.8)	83.0 (±14.79)
Physical health	75.44 (±22.1)	84.41 (±17.26)
Emotional functioning	71.79 (±10.6)	80.86 (±19.64)
Social functioning	77.14 (±31.8)	87.42 (±17.18)
School functioning	76.5 (±14.1)	78.63 (±20.53)

**Table 3:** SDQ Mean scores of children with heart disease and CPR in comparison with healthy cohort from the LifeChild Study [16].

	Children with Heart Disease	Healthy Cohort	Limit Values		
**Behavioral strengths and difficulties parent reports**					
	n = 6	n = 1186	normal	borderline	abnormal
Total score	13.5 (±3.0)	n/a	0–13	14–16	17–40
Prosocial behavior	8.6 (±2.2)	7.91 (±1.89)	6–10	5	0–4
Hyperactivity	5.8 (±0.7)	3.74 (±2.43)	0–5	6	7–10
Emotional symptoms	2.6 (±1.8)	1.11 (±1.45)	0–3	4	4–10
Conduct problems	1.3 (±1.4)	2.05 (±1.56)	0–2	3	4–10
Peer relationship problems	2.0 (±0.7)	1.11 (±1.45)	0–2	3	4–10
**Behavioral strengths and difficulties self-reports**					
	n = 8	n = 1042			
Total score	11.75 (±3.0)	n/a	0–15	16–19	20–40
Prosocial behavior	7.4 (±2.1)	7.88 (±1.80)	6–10	4	0–4
Hyperactivity	4.0 (±0.7)	3.46 (±2.26)	0–5	6	7–10
Emotional symptoms	3.75 (±2.1)	2.47 (±2.17)	0–5	6	7–10
Conduct problems	1.9 (±1.4)	1.55 (±1.37)	0–3	4	5–10
Peer relationship problems	3.1 (±0.8)	2.05 (±1.63)	0–3	4	5–10

## Data Availability

The original contributions presented in this study are included in the article. Further inquiries can be directed to the corresponding authors.

## Appendix A

### Appendix A.1. First Data Query via ICD or OPS Codes

1. ICD-Codes:

- R09.2 Respiratory arrest, including cardiopulmonary arrest
- I46. Cardiac arrest, I46.0 successful, I46.9 without successful resuscitation
- I49.0 Ventricular flutter and fibrillation
- R00.3 Pulseless electrical activity, excl. cardiac arrest
- R57.9 Peripheral circulatory failure

### Appendix A.2. -T98.-Consequences of Other and Unspecified Effects of External Causes, Consequences of Amputation n.e.c. Consequences of Resuscitation

2. OPS codes:

- 8–779 Measures in the context of resuscitation
- 8–771 Cardiac or cardiopulmonary resuscitation
- 8–772 Operative resuscitation

### Appendix A.3. Second Data Query Using Keywords from the Diagnosis List and the Epicrisis

- Post-resuscitation
- Post cardiopulmonary resuscitation
- Post-circulatory arrest
- Post-cardiac arrest
- Post-cardiac massage
- After resuscitation measures
- Exitus letalis
